# Impact of the Covid‐19 pandemic on neuropsychiatric symptoms and antipsychotic prescribing for people with dementia in nursing home settings

**DOI:** 10.1002/gps.5878

**Published:** 2023-01-27

**Authors:** Joanne McDermid, Clive Ballard, Zunera Khan, Dag Aarsland, Chris Fox, Jane Fossey, Linda Clare, Esme Moniz‐Cook, Maria Soto‐Martin, Adrienne Sweetnam, Kathryn Mills, Jeffrey Cummings, Anne Corbett

**Affiliations:** ^1^ University of Exeter Medical School University of Exeter Exeter UK; ^2^ Institute of Psychiatry, Psychology and Neuroscience King's College London London UK; ^3^ NIHR Applied Research Collaboration South‐West Peninsula Exeter UK; ^4^ Faculty of Health Sciences University of Hull Hull UK; ^5^ Centre Hospitalier Universitaire de Toulouse Toulouse France; ^6^ Chambers‐Grundy Center for Transformative Neuroscience Department of Brain Health School of Integrated Health Sciences University of Nevada Las Vegas (UNLV) Las Vegas Nevada USA

**Keywords:** antipsychotics, Covid, dementia, neuropsychiatric, nursing home

## Abstract

**Objectives:**

This study aimed to determine the impact of the Covid‐19 pandemic on neuropsychiatric symptoms and antipsychotic use in people with dementia living in nursing homes.

**Methods:**

This was a comparative analysis of baseline data from two large nursing home studies, one conducted during (COVID‐iWHELD study) and one prior (WHELD study) to the pandemic. It involves data from 69 and 149 nursing homes, and 1006 and 666 participants respectively. Participants were people with established dementia (score >1 on Clinical Dementia Rating Scale). Resident data included demographics, antipsychotic prescriptions and neuropsychiatric symptoms using the Neuropsychiatric Inventory Nursing Home version. Nursing home data collected were nursing home size and staffing information.

**Results:**

Overall prevalence of neuropsychiatric symptoms was unchanged from pre‐pandemic prevalence. Mean antipsychotic use across the sample was 32.0%, increased from 18% pre‐pandemic (Fisher's exact test *p* < 0.0001). At a nursing home level, the medians for the low, medium and high tertiles for antipsychotic use were 7%, 20% and 59% respectively, showing a disproportionate rise in tertile three. Residents in these homes also showed a small but significant increase in agitation.

**Conclusion:**

There has been a significant increase in antipsychotic prescribing in nursing homes since the COVID‐19 pandemic, with a disproportionate rise in one third of homes, where median prescription rates for antipsychotics were almost 60%. Strategies are urgently needed to identify these nursing homes and introduce pro‐active support to bring antipsychotic prescription rates back to pre‐pandemic levels.

## Key points

- The Covid‐19 pandemic has presented major challenges to the care home sector, raising the risk of worsening neuropsychiatric symptoms and antipsychotic prescribing practice
- Comparison of two care home samples indicates no overall change in neuropsychiatric symptoms but an 18% increase in antipsychotic prescribing
- The increase in antipsychotic use occurred disproportionately in one third of the 149 homes included in this study
- Most nursing homes have weathered the Covid‐19 pandemic and have not seen increases in use of harmful antipsychotic drugs in residents with dementia but some homes do need help to get back on track

## 1 INTRODUCTION

The unnecessary use of antipsychotic medications in people with dementia has been a major issue in research, policy and practice over recent decades. Historically used widely to manage the neuropsychiatric symptoms of dementia such as agitation, aggression and psychosis, atypical antipsychotics confer only modest clinical benefit when used in the short‐term. Meta‐analyses of clinical trials indicate borderline statistical significance for clinical benefit to aggression following treatment with risperidone,^1^ but not for agitation or psychosis. The benefit is even less clear with other antipsychotics such as quetiapine and importantly, many trials reported high placebo response which further limits the benefits reported.^2^ This marginal benefit is even less favourable when considered in the context of the well‐established harms associated with these medications. A review of 15 trials in people with AD showed a significant mortality risk following treatment with these medications, and studies focusing on the withdrawal of atypical antipsychotics have reported a reduction in this risk following halting of prescriptions.^3^ Additional important adverse effects include a clinically significant increase in cognitive decline, a three‐fold increase in cerebrovascular events such as stroke, as well as extra‐pyramidal symptoms, peripheral oedema, sedation, prolonged Q‐Tc interval, infections and abnormal gait.^4^ These effects worsen with longer‐term use, and trials of antipsychotics lasting 6 months or more show mortality rates of up to 59%.^2^, ^5^, ^6^ These harms are particularly prevalent in nursing home settings, where many people have late‐stage dementia with complex co‐morbidities, including a high prevalence of neuropsychiatric symptoms. Prescription rates of antipsychotics for people with dementia in nursing homes exceeded 45% in the early 2000s.^7^ This led to high profile initiatives across the US, Europe and elsewhere which have successfully reduced antipsychotic prescribing to less than 20% of nursing home residents,^8^, ^9^ as part of programmes internationally leading to a reduction of the prescription of antipsychotics for people with dementia. For example, in the UK antipsychotic prescription for people with dementia was reduced by 50% nationally between 2009 and 2015.^10^, ^11^ These reductions were achieved by clinicians adopting updated prescribing and treatment guidelines,^12^, ^13^ combined with an increased focus on person‐centred care and use of non‐drug psychosocial interventions as a first‐line measure.^7^, ^9^ The latter not only reduce antipsychotic use but are also associated with benefit to quality of life, neuropsychiatric symptoms, apathy and pain.^7^, ^9^

The Covid‐19 pandemic represents a considerable potential risk to the positive changes that were achieved prior to 2020. During the pandemic, social care systems worldwide experienced an unprecedented level of burden amongst frail, vulnerable residents. There were severe shortages of staff due to ill health and the social restrictions and society‐wide lockdowns prevented families from visiting and had marked effects on residents.^14^, ^15^ Preliminary studies have reported accelerated cognitive decline and an increase in depression, anxiety and neuropsychiatric symptoms in people with dementia in the community during the pandemic.^16^, ^17^, ^18^ In addition, one study reported a 10% rise in antipsychotic use from March to July 2020 in people with dementia and this observation has been supported by further reports.^19^ However, there has been very little focus on people with dementia living in nursing homes.^20^, ^21^ This leaves key questions unanswered, such as the impact of the pandemic on neuropsychiatric symptoms and antipsychotic prescribing amongst these individuals.^22^ The potential adverse effects associated with atypical antipsychotics may be of particular concern in the context of the COVID pandemic, where mortality and morbidity rates are already increased. Here we report an analysis of neuropsychiatric symptoms and antipsychotic prescribing rates across 149 UK nursing homes during the Covid‐19 pandemic and compare the findings with a baseline dataset collected for a clinical trial conducted prior to the pandemic. The two datasets were collected using closely similar methodologies and settings, thus allowing for a unique insight into the impact of the pandemic on these key indicators of health and wellbeing in nursing homes.

## 2 METHODS

Study Design: This is an analysis of baseline data from 666 participants from 149 nursing homes taking part in the COVID‐ Improving Well Being and Health in People with Dementia (COVID‐iWHELD) nursing home study conducted in the UK in 2020–2022 (ClinicalTrials.gov Ref: NCT04590469). The study received ethical approval from the West Midlands Coventry & Warwick National Health Service (NHS) Research Ethics Committee (Ref: 20/WM/0289). The data is compared to published data from the Improving Well Being and Health in People with Dementia (WHELD) study conducted in 2015–2016 (ClinicalTrials.gov Ref: NCT01855152) which received ethical approval from the Oxford C NHS Research Ethics Committee (Ref: 13/SC/0281). The full WHELD protocol is published elsewhere.^23^ For both studies, all nursing homes providing care for people with dementia were eligible unless they were under special measures relating to inspection by the UK Care Quality Commission (CQC), to make the sample of homes as representative as possible. Eligible residents were identified and approached by nursing home staff, after which consent was obtained from residents, with the involvement of a consultee where capacity was lacking.

Selection of participants: For the WHELD study sample all residents were considered potentially eligible for inclusion if they met criteria for dementia (defined as a score 1 or greater on the Clinical Dementia Rating [CDR],^26^ operationalized to require a minimum level of cognitive, functional, and neuropsychiatric features). The study sought to recruit nine participants per nursing home. In total 1396 potential participants were identified. Consent was obtained for 1002 of these participants, of whom 124 had a CDR of 0 or 0.5 and were excluded. Consent was obtained from a legal representative for each participant using the iWHELD digital information and consent portal. This occurred after the legal representative had the opportunity to review the study information and discuss it with a member of the iWHELD team using a virtual communication platform (Zoom or TEAMS). Recruitment was open from January 2013 to September 2015. Detailed information was collected for five residents per nursing home based on the power calculation for the randomized cluster trial of the iWHELD intervention in this sample. A larger sample per participating nursing home would have reduced power within the context of the cluster trial design. Participants had a diagnosis of dementia, fulfilling a score of 1.0 or greater in the CDR Scale^24^ or a FAST score of ≥ 4,^25^ and were residing in a participating nursing home. 785 potential participants were identified, of whom 745 were consented to participate. All had a CDR ≥1 and/or a FAST≥ 4, but full information regarding antipsychotic prescribing was only available for the 666 of the residents who were recruited to the study. In addition to the 666 iWHELD participants (based on approximately five participants per care home), data regarding antipsychotic medication was available at a care home level for all residents with a recorded diagnosis of dementia according to ICD 10 in the nursing home notes across the participating homes. The study was conducted between April 2021 and January 2022.

Data Collection and Analysis: Baseline assessments collected demographic information and the use of antipsychotics from prescribing records. Neuropsychiatric symptoms were assessed by informant interview using the Neuropsychiatric Inventory (Nursing Home version),^26^ with care staff as the informants. Data were collected from each home to capture the home size (number of beds), number of residents with dementia, and staff profile (number of agency shifts, sick days due to COVID‐19 and total sick days in the last month). Descriptive statistics were created and a between‐group analysis conducted using an analysis of variance (ANOVA) to explore the impact of home characteristics and neuropsychiatric symptoms on antipsychotic frequency. Antipsychotic use from the iWHELD study was compared with data from the earlier WHELD nursing home study with comparable methodology and using the same eligibility criteria conducted in the UK in 2015–2016 (ClinicalTrials.gov Ref: NCT01855152) to provide a direct comparator for pre‐ and post‐pandemic usage.

## 3 RESULTS

Demographic information about both study samples is shown in Table 1. Antipsychotic data were available for 3794 residents with an ICD 10 diagnosis of dementia based on nursing home records. 1060 participants were approached, for whom legal representatives gave consent for 707 individuals. 40 of these individuals withdrew before baseline assessment, and data was incomplete, giving a cohort of 666 individuals up to December 2021, who were the analysis cohort for the current study. More detailed data were collected for the 666 residents who were registered as iWHELD study participants. This sample of 666 residents was 69.5% female with a mean age of 85.5 (SD 8.0). In this group the overall prevalence of delusions (20.3%), hallucinations (15.3%) and agitation (46.6%) was consistent with pre‐pandemic levels (Table 2). Mean antipsychotic frequency in the whole nursing home population was 33% (*n* = 1252) amongst all residents with dementia in the participating nursing homes, and 32.0% in the iWHELD study sample (*n* = 213). This represents a significant increase from 18% (*n* = 181) in the WHELD sample pre‐pandemic (Fisher's Exact Test *p* < 0.0001, Table 3). Antipsychotics were prescribed to 65 (53.6%) of individuals with clinically significant psychosis (≥6 on combined NPI scores for delusions and hallucinations) and 121 (54.8%) of the people with clinically significant agitation (NPI agitation score ≥3), compared to 61 (15.8%) of people with neither of these symptoms at a clinically significant threshold. 62 people had both clinically significant psychosis and agitation.

**TABLE 1:** Cohort characteristics in both samples

	iWHELD (pan pandemic) *N* = 666	WHELD (pre‐pandemic) *N* = 847
Age	85.5 (SD 8.0).	88.4 (SD 8.5)
Female gender	463 (69.5%)	586 (69.2%)
Dementia severity		
Mild/moderate	186 (27.9%)	159 (18.8%)
Moderately severe/severe	480 (72.1%)	688 (81.2%)

**TABLE 2:** Neuropsychiatric symptoms in residents across the COVID‐iWHELD study sample (pandemic period April 2021‐January 2022) and the WHELD study sample (pre‐pandemic period 2015‐2016), showing incidence of symptoms across the three antipsychotic frequency groups in the COVID‐iWHELD sample

	COVID‐iWHELD study sample (*n* = 666)										Pre‐pandemic WHELD study sample^9^ (*n* = 1006)
Neuropsychiatric symptoms	Low antipsychotic use (*N* = 224)			Medium antipsychotic use (*N* = 225)			High antipsychotic use (*N* = 217)			Total sample)	
	*N* (%)	Mean domain score	SD	*N* (%)	Mean domain score	SD	*N* (%)	Mean domain score	SD		
Delusions	43 (19.2)	0.79	2.23	40 (17.7)	0.40	1.56	49 (22.6)	1.47	1.56	132 (19.8%)	171 (17.6%)
Hallucinations	35 (15.6)	0.98	2.48	28 (12.4)	1.05	2.61	40 (18.4)	1.50	3.20	103 (15.5%)	146 (14.5%)
Agitation	99 (44)	2.10	3.01	101 (44.7)	2.31	3.27	110 (50.7)	2.88	3.59	310 (46.5%)	583 (50.8%)

**TABLE 3:** Nursing home characteristics across the three antipsychotic frequency groups in the COVID‐iWHELD study sample (Pandemic period, April 2021‐January 2022)

	Low antipsychotic use (*n* = 44 nursing Homes)		Medium antipsychotic use (*N* = 46 nursing homes)		High antipsychotic use (*N* = 43 nursing homes)	
	Mean	SD	Mean	SD	Mean	SD
Total number of residents	40.4	27.1	44.4	27.4	42.49	36.5
Number of residents with dementia	25.0	19.1	27.1	16.2	22.19	13.7
Number of temporary staff shifts	11.49	33.4	12.5	28.4	18.38	47.6
Staff sick days due to COVID in last month	3.98	7.7	6.8	12.4	2.81	6.2
Total staff sick days in last month	28.45	93.03	35.17	76.3	17.4	39.0

Nursing homes were grouped into tertiles according to the percentage of residents with an antipsychotic prescription (Low: 0%–14%, Medium: 14%–36%, High: >36%). At a nursing home level, the median frequency of prescriptions was 7%, 20% and 59% respectively across the low, medium and high antipsychotic use tertiles, highlighting a disproportionate increase in at least one‐third of nursing homes. Comparatively, in the pre‐pandemic study all homes had prescribing rates of six to 33%, with only two outliers with rates of 40% and 52% respectively, indicating a far lower overall prescribing rate and less variability pre‐pandemic.

To further investigate this substantial rise in antipsychotic prescriptions in tertile 2 and 3, we examined the potential associations with nursing home factors and neuropsychiatric symptoms. There were no significant differences in nursing home size, the number of residents with dementia, sick days or use of temporary staff across the three groups (Table 3). The ANOVA showed a significant but modest elevation in levels of agitation and delusions in nursing homes with higher antipsychotic use (*F* = 3.23; *p* = 0.04 and *F* = 4.09; *p* = 0.017 respectively) (Table 2).

## 4 DISCUSSION

This study provides the first direct insight into the impact of the COVID‐19 pandemic on neuropsychiatric symptoms and antipsychotic use in people with dementia living in nursing homes. These are key indicators of care quality and resident wellbeing, and a critical driver of overall health in nursing home residents. The study includes data from two large nursing home trial datasets, one collected prior to the pandemic and involving 69 nursing homes and 1006 participants, and one collected during the pandemic and involving 149 nursing homes and 666 participants. This has allowed for direct comparison of the two time periods, enabling conclusions to be drawn about the likely impact of the pandemic on prescribing practices. Whilst the overall number of participants treated with antipsychotics rose from 18% to 32% (55% increase), this was driven to a large extent by a disproportionate increase in antipsychotic prescriptions in one third of the homes sampled where the median prescription rate per residents of antipsychotics was 59%, exceeding even the levels of prescription in the early 2000s.

It should be acknowledged however, that given the considerable strain and burden experienced by the nursing homes during the pandemic, it is an impressive example of resilience within the sector and the long‐term success of cultural change in both care quality and prescribing practice that many nursing homes maintained antipsychotic prescriptions at pre‐pandemic levels. It is important to recognise and applaud this achievement. This provides a strong rationale for the continued focus on investment in staff training in person‐centred care and management of neuropsychiatric symptoms, including tailoring of care plans to include protected time for social interaction and pleasant activities. Outcomes from major trials of psychosocial interventions in nursing homes report improvements to staff burden, perceived skills and confidence following structured training,^27^ and it is likely that these benefits contributed to an increased resilience across the majority of the workforce during the challenges imposed by the pandemic.

However, in the remaining 32% of nursing homes where antipsychotic prescribing rose to 59% there is a considerable challenge moving forward. The reasons for the increase in prescribing within these settings is not clear. There was no significant association with key nursing home characteristics such as home size, resident numbers, staffing structure or staff absences. The analysis did however show a modest increase in neuropsychiatric symptoms in residents in these homes, particularly in agitation and delusions. It is likely that this uptick in symptoms resulted, in part, in the increased prescribing rate, although the reason for symptom emergence and worsening is itself unclear. Whilst it is reassuring that the main increase in prescribing occurred in people with clinically significant neuropsychiatric symptoms, the limitations exerted by the pandemic on provision of alternative interventions, combined with the challenges of monitoring symptoms during this time may have led to more rapid prescribing in these groups and the training and support processes available in participating care homes were not captured and so no conclusions can be drawn on the influence of existing staff training and knowledge in these homes. However, it will be essential to proactively identify these nursing homes that have experienced an increase in prescribing and ensure they are fully supported in their institutional recovery from the pandemic, including ensuring evidence‐based training and support is fully available in these settings. Programmes such as the evidence‐based WHELD training package will be valuable for embedding this practice across settings. These findings also further emphasise the need for a proactive strategy to revisit antipsychotic prescribing guidance in order to prevent resurgence of use back to pre‐pandemic levels. These efforts will be essential to ensure health and wellbeing outcomes for people with dementia stabilise and continue to improve, and to avoid any polarisation in care quality across the social care sector.

This study provides a unique insight into the impact of the COVID19 pandemic on prescribing practice and neuropsychiatric symptoms amongst people with dementia in nursing homes. However, we acknowledge some key limitations. The datasets used for comparison pre‐ and post‐pandemic are from separate studies and so do not allow for a direct comparison within nursing homes. It should also be noted that care homes under CQC special measures, who may have been expected to be under even greater pressures during the pandemic were excluded from the study, which may potentially have underestimated overall increases in antipsychotic prescribing. It is also unclear what the drivers for change in prescribing practice were in the homes included in this study, particularly since no data was captured pertaining to the prescriber themselves. It should be noted though that the pandemic restrictions in the UK did limit the capacity for review by specialist old age psychiatrists, other members of the community mental health teams and primary care teams, and most reviews were conducted virtually. These limitations for medical review and assessment may also have been a contributory factor in the increased rates of prescribing. However, the data presented does provide a novel and important snapshot of prescribing practice during a pivotal moment for healthcare. The data is derived from robust, large‐scale clinical trials, indicating a high level of data quality and scrutiny.

Overall the outputs of this study have considerable import for shaping the future of policy in this field, as well as highlighting the strengths of resilience and care practice that are already in place within the care sector. It will be critical to build on these aspects to ensure any discrepancies in resource and training availability do not drive differential trends within the nursing home sector, and to build policy to ensure all homes are supported in providing high quality care to achieve and maintain low levels of antipsychotic prescribing.

## CONFLICT OF INTEREST

Clive Ballard has received consulting fees from Acadia pharmaceutical company, AARP, Addex pharmaceutical company, Eli Lily, Enterin pharmaceutical company, GWPharm, H.Lundbeck pharmaceutical company, Novartis pharmaceutical company, Janssen Pharmaceuticals, Johnson and Johnson pharmaceuticals, Novo Nordisk pharmaceutical comapny, Orion Corp pharmaceutical company, Otsuka America Pharm Inc, Sunovion Pharm. Inc, Suven pharmaceutical company, Roche pharmaceutical company, Biogen pharmaceutical company, Synexus clinical research organization and tauX pharmaceutical company and research funding from synexus clinical research organization, Roche pharmaceutical company, Novo Nordisk pharmaceutical company, Novartis pharmaceutical company, Medical research council (UK), Wellcome trust (UK), National Institute for Health Research (UK), National Institute for Health (US), IMI (Eu), Michael J Fox foundation (US), Alzheimer's Disease Drug Discovery foundation (US), Alzheimer's Society (UK), Parkinson's Society (UK), Alzheimer's Research UK, the Gilling's foundation and BRACE (UK). Jeffrey Cummings has provided consultation to Acadia, Alkahest, AlphaCognition, AriBio, Biogen, Cassava, Cortexyme, Diadem, EIP Pharma, Eisai, GemVax, Genentech, Green Valley, Grifols, Janssen, Lilly, LSP, Merck, NervGen, Novo Nordisk, Oligomerix, Ono, Otsuka, PRODEO, Prothena, ReMYND, Resverlogix, Roche, Signant Health, Suven, and United Neuroscience pharmaceutical, assessment, and investment companies. Jeffrey Cummings is supported by NIGMS grant P20GM109025; NINDS grant U01NS093334; NIA grant R01AG053798; NIA grant P20AG068053; NIA grant R35AG71476; Alzheimer's Disease Drug Discovery Foundation (ADDF); and the Joy Chambers‐Grundy Endowment. Anne Corbett has provided consultation to Acadia, Addex, Novartis, Janssen and Suvonion pharmaceutical companies. Joanne McDermid, Zunera Khan, Dag Aarsland, Chris Fox, Jane Fossey, Linda Clare, Esme Moniz‐Cook, Maria Soto‐Martin, Adrienne Sweetnam, Kathryn Mills report no financial relationships with commercial interests.

## Data Availability

The data that support the findings of this study are available from the corresponding author upon reasonable request.
